# Bart's syndrome associated with a disorder of sexual differentiation: An atypical presentation in a Cameroonian newborn

**DOI:** 10.1002/ccr3.5234

**Published:** 2022-01-07

**Authors:** Odette Berline Sigha, Ritha Mbono Betoko, Grace Anita Nkoro, Mireille Fossi Happi, Charlotte Eposse Ekoube, Benjamin Bertrand Kelbaba, Edgar Mandeng Ma Linwa, Emmanuel Armand Kouotou

**Affiliations:** ^1^ Faculty of Health Sciences University of Bamenda Bambili Cameroon; ^2^ Service de dermatologie Hôpital Laquintinie de Douala Douala Cameroun; ^3^ Département de pédiatrie Hôpital Laquintinie de Douala Douala Cameroun; ^4^ Faculté de Médecine et des Sciences Pharmaceutiques Université de Douala Douala Cameroun; ^5^ Service de dermatologie Hôpital Gynéco‐obstétrique et Pédiatrique de Yaoundé Yaoundé Cameroun; ^6^ Faculté de Médecine et des Sciences Biomédicales Université de Yaoundé 1 Yaoundé Cameroun; ^7^ Service de cardiologie Hôpital Laquintinie de Douala Douala Cameroun; ^8^ Faculty of Health Sciences University of Buea Buea Cameroon; ^9^ Service de dermatologie Centre Hospitalier Universitaire de Yaoundé Yaoundé Cameroun

**Keywords:** Bart's syndrome, Cameroon, disorder of sexual differentiation

## Abstract

Bart's syndrome consists of congenital aplasia of the skin affecting only the lower limbs, associated with bullae over the skin and/or mucous membranes, as well as a nail anomaly. It is an extremely rare genetic disorder, which can be associated with other birth defects. We report the case of a newborn baby admitted at day 0 of life in the neonatal department, for multifocal skin detachment predominantly at the lower limbs. In addition, examination of the external genitalia revealed a clitoridomegaly genital bud measuring 14 mm, scrotalized and unfused genital bulges with the presence of 2 orifices. No gonad was palpated. The clinical diagnosis of Bart's syndrome associated with a disorder of sexual differentiation was retained. We hereby report the first case of Bart's syndrome described in Cameroon in association with a disorder of sexual differentiation.

## Graphical abstract

Bart's syndrome which is an extremely rare genetic disorder, consists of congenital aplasia of the skin affecting only the lower limbs, associated with bullae over the skin and/or mucous membranes. It can be associated with other birth defects. We hereby report the first association of Bart’s syndrome with disorder of sexual differentiation. This case is interesting because this clinical situation has not yet been described in literature. It may lead to increased awareness of caregivers about this particular presentation.

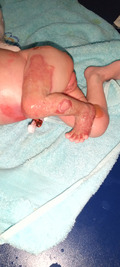

## 1 INTRODUCTION

Congenital skin aplasia also called aplasia cutis congenita (ACC) is a rare birth defect characterized by a localized absence of skin and in some cases subcutaneous tissue.^1^ The first case of ACC was described in 1767 by Cordon.^2^ This is a very rare disease. Until 2013, only around 500 cases were reported worldwide^2^ and all outside Africa. There is no racial or sexual predisposition. Globally, the estimated incidence of ACC is 3 in 10,000 live births.^2^

The diagnosis is clinical and may prove difficult due to the polymorphism and variable localization of the skin lesions.^3^ In 65%–85% of cases, the lesions affect the scalp. The other locations represent 15%–35% of cases.^4^

FRIEDEN in 1986 created a classification system for ACC consisting of nine groups based on the number and location of lesions, the presence or absence of associated malformations and hereditary character.^4^ ACC type 6 is a genodermatosis characterized by a triad of clinical manifestations: ACC affecting the lower limbs, epidermolysis bullosa, and sometimes nail abnormalities. This clinical trial is also called Bart's syndrome, first described by Bruce J. Bart in 1966.^3^, ^5^

In more severe cases of Bart's syndrome, especially those associated with junctional epidermolysis bullosa, the patient may have other birth defects such as pyloric atresia, ureteral stenosis, renal abnormalities, development rudimentary ear, and orbital hypertelorism.^3^ Given the rarity of this syndrome, it is very likely that this list of abnormalities is not exhaustive.

In sub‐Saharan Africa, we have identified five cases of ACC affecting the lower limbs, including two cases of ACC type 6 or Bart's syndrome, respectively, in Nigeria and Côte d'Ivoire^6^, ^7^ and three cases of typical ACC type 7 in Nigeria.^8^, ^9^

We report the specific case of a newborn with Bart's syndrome associated with a disorder of sexual differentiation (DSD). Few data are found in the literature concerning such an association.

## 2 OBSERVATION

We describe a newborn baby admitted at day 0 of life in the neonatology department, for multifocal skin detachment observed in the delivery room. He was born to a mother after a well‐followed pregnancy. Tetanus, antianemic and antimalarial prophylaxis had been administered to the mother. No notion of traditional self‐medication, hospitalization, maternal fever, herpes simplex infection, or chickenpox was reported. In addition, the two obstetric ultrasounds performed during pregnancy found an evolving single‐fetal intrauterine pregnancy. No notion of consanguinity or genodermatosis was detected in the parents. Our patient, second sibling, was born at term vaginally to a 21‐year‐old mother. The first child was born at term without any abnormalities noted at birth and was 2 years old. Neonatal adaptation was good, with an Apgar score of 8–9–10. The birth weight was 2300 g, the height 49 cm and the head circumference 33 cm. These anthropometric parameters were below the 10th percentile for gestational age. This corresponds to a symmetrical intrauterine growth restriction. There was no notion of resuscitation at birth, and the amniotic fluid was clear.

On clinical examination, we noted a good general state, colored conjunctiva and no icterus, temperature at 36.6°C, heart rate at 135 beats/minute, and respiratory rate at 36 cycles/minute.

**FIGURE 1:**
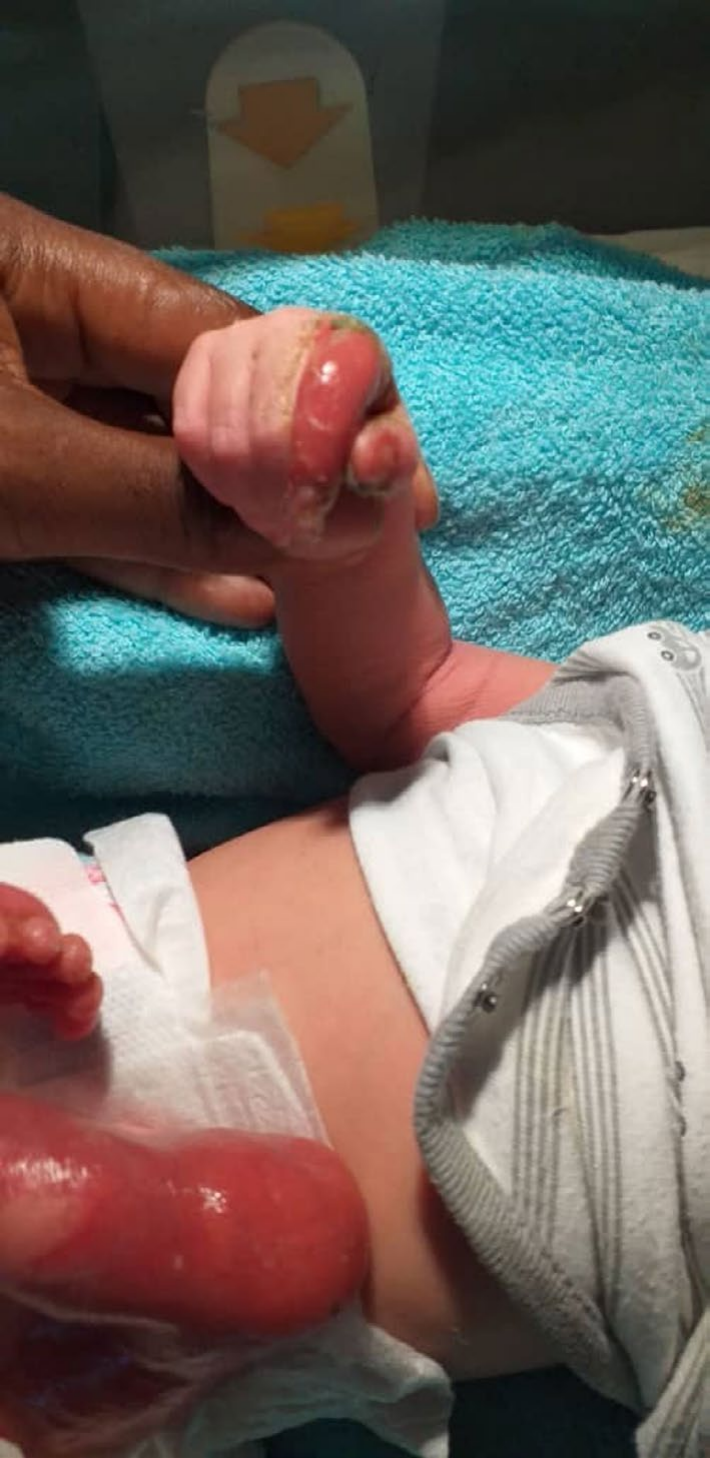
Cutaneous aplasia of the left lower limb and post‐bullous lesion of the right hand

**FIGURE 2:**
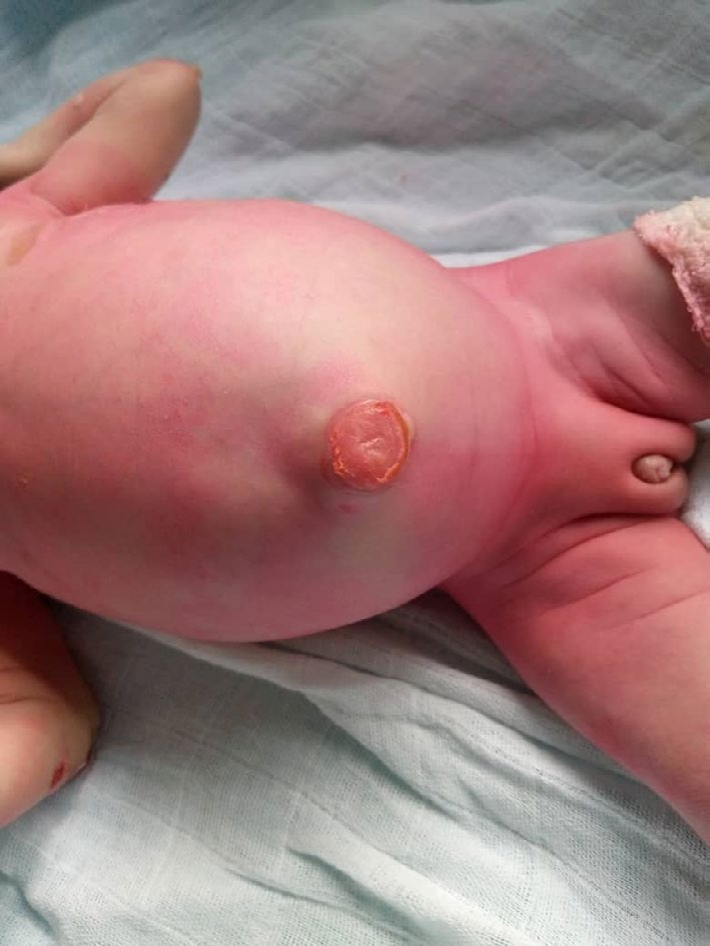
Umbilical ulceration and sexual differentiation anomaly

On skin examination at birth, aplastic skin was noted extending from the dorsal aspect of the foot to the outer lateral aspect of the left thigh and at the level of the umbilicus; post‐bullous erosions were clearly seen at the right thumb, right index finger, and dorsal aspect of the left hand; no onychodystrophy was noted (Figure 1). In addition, examination of the external genitalia revealed a genital bud measuring 14 mm, scrotalized and unfused genital bulges with the presence of 2 orifices (Figure 2). No gonad was palpated. The cardiovascular examination performed was normal; a capillary refill time (CRT) of less than 3 s, the different pulses perceived, and heart sounds well perceived at the different auscultation foci without added sounds. The rest of the clinical examination was unremarkable.

On the 3rd day of hospitalization, there was a gradual appearance of bullae particularly in the left knee, right buttock, right upper limb, and chest. These bullae quickly gave way to erosions.

Based on this clinical picture combining a predominant cutaneous aplasia in the lower limbs, the presence of bullae and post‐bullous lesions of spontaneous appearance as well as abnormal genitalia, the clinical diagnosis of Bart's syndrome associated with a disorder of sexual differentiation was made. It was more likely to be a 46XX DSD due to clitoridomegaly and no palpable gonads.

The blood tests requested at birth; the blood count, total and direct bilirubin, serum urea, and serum creatinine were normal. Furthermore, a high C‐reactive protein to 24 mg/L was observed. The histopathological study of a bullous lesion and an abdomino‐pelvic ultrasound to detect any associated malformations and describe the type of internal genitalia were not carried out because of financial constraints. In addition, karyotyping, not available in our country, could not be made.

**FIGURE 3:**
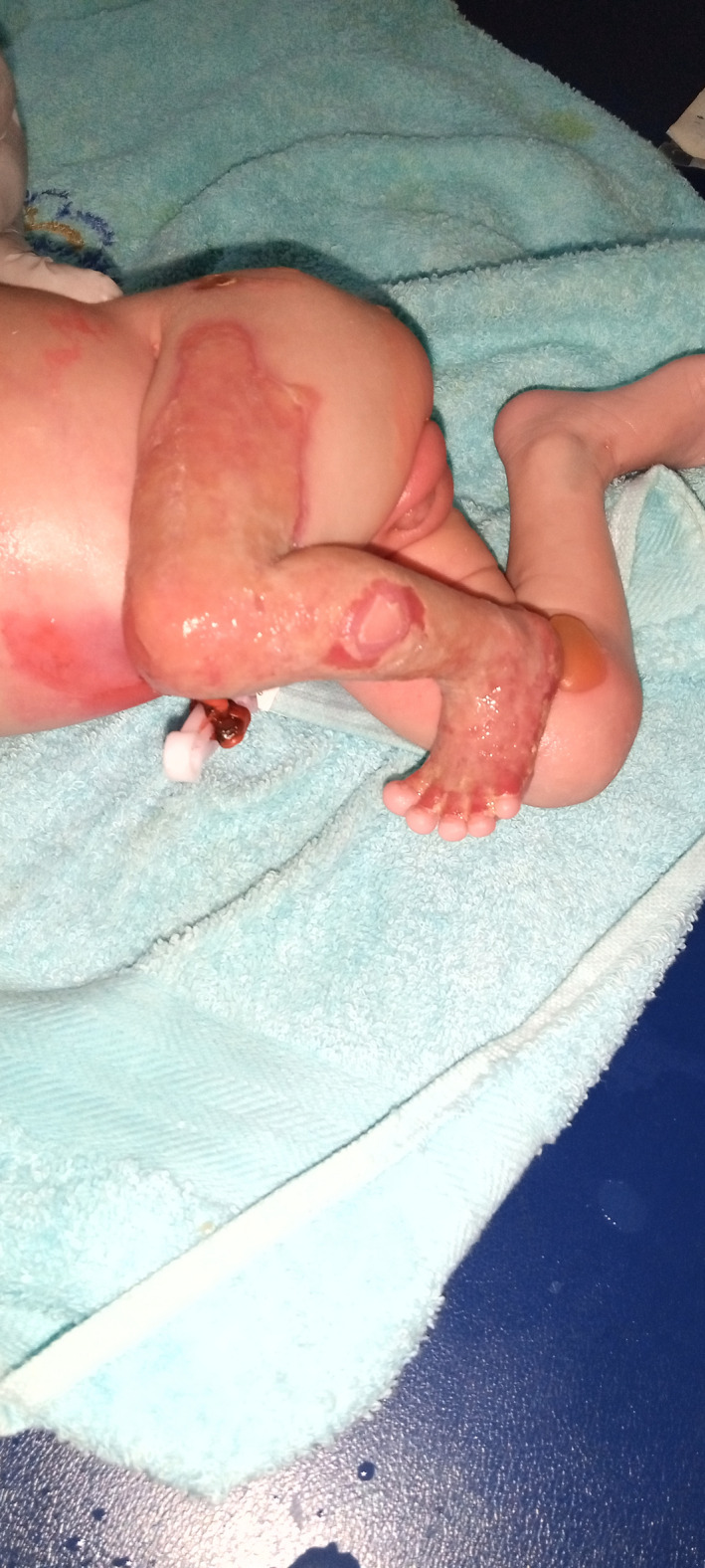
Start of formation of granulation tissue over the left lower limb and right knee bullae

The management of the newborn consisted of broad‐spectrum antibiotics, parenteral rehydration, analgesia, and treatment of ulcerations (dressing based on trolamine, polyvidone iodine, paraffin tulle, after cleaning with an antiseptic). At 1 week of hospitalization, the appearance of granulation tissue over the lesion in the right leg was observed. On Day 19 of hospitalization, the patient was discharged by the family against medical advice but nevertheless continued the dressings on an outpatient basis. A month after discharge, we noted an almost complete wound healing (Figure 3); but a week later, the patient was re‐admitted at the emergency room for generalized pallor and dyspnea. The patient died few hours later in a clinical context of severe anemia (Hemoglobin level of 3 g/dl).

## 3 DISCUSSION

Bart's syndrome is an extremely rare genetic disorder. It is characterized by congenital localized absence of skin (affecting only the lower limbs), bullous skin lesions and/or mucous membranes and sometimes nail disorders, including congenital absence or nail dystrophy.^3^

In Bart's syndrome, ACC is usually unilateral.^10^ In our case, only the left leg was affected and we did not notice any nail abnormality. ACC is usually sporadic, but autosomal dominant and less frequently, autosomal recessive cases have been reported.^11^ Our case was probably sporadic as there was neither consanguinity between the patient's parents nor a family history of a similar lesion.

The exact pathogenesis of ACC remains poorly understood, but etiologies including fetus papyraceus, amniotic band syndrome, chromosomal abnormalities, drugs (methimazole, carbimazole, and valproic acid), toxins (cocaine), intrauterine trauma, and congenital infections caused by herpes simplex virus and varicella‐zoster virus have been suggested to explain this disease.^6^ No apparent cause could be found in our case, because the mother did not report any drug exposure nor herpes or chickenpox type lesions during the pregnancy.

Bart's syndrome is usually diagnosed based on the clinical presentation and skin biopsy analysis, which helps to determine the type of epidermolysis bullosa. A genetic study is usually done to look for the exact genetic mutation that could confirm the final diagnosis.^3^, ^11^ In our case, because of financial means, a histopathological examination of the bullous lesions could not be carried out. Nevertheless, we have discussed the diagnosis based on the clinical presentation.

In more severe cases of Bart's syndrome, particularly those associated with junctional epidermolysis bullosa, the patient may present with other birth defects.^3^ In our case, the child also presented another congenital defect. It was a disorder of sexual differentiation, probably a 46 XX DSD. This presumptive diagnosis was evoked because of abnormal genitalia without palpable gonads. This association was not yet reported in previous studies.

Disorders of sexual differentiation are a group of congenital conditions with atypical chromosomal, gonadal, or phenotypic sex. These conditions may be associated with variations in developmental programming genes and hormones.^12^ The incidence of this condition is estimated to 1: 2000–1: 4500.^12^, ^13^

The embryonic differentiation begins at the 6th week after fertilization. Any abnormality occurring during sexual differentiation including genetic mutations and chromosomal abnormalities can be the cause of abnormal genitalia.^12^ Abnormalities of sexual differentiation can be associated with other malformative syndromes. Thus, the child should be carefully examined for other abnormalities.^14^ When faced with a congenital defect of the external genitalia, many investigations must be initiated in order to decide on the overall care of the child. These investigations include the study of the internal genital anatomy (pelvic ultrasound, MRI, endoscopy, laparoscopy), the study of the karyotype, and endocrine explorations (assay of 17‐OH progesterone, testosterone DHT, and D4 androstenedione).^12^

Regarding the management of Bart's syndrome, conservative treatment by local wound care is generally recommended. Some very extensive lesions may require subsequent surgery, including skin grafts.^3^, ^8^ Close follow‐up is necessary in order to avoid superinfection and to prevent and treat serious complications such as bleeding, infection, hypothermia, and hypoglycemia.^15^

Our patient benefited from a local treatment based on dressings with trolamine, povidone iodine, and paraffin tulle. One week after treatment, the ulcerations were covered by granulation tissue, and 1 month after discharge, almost all the lesions had healed. Despite the good clinical progress of the skin lesions, our patient died from severe anemia, probably due to bleeding from the lesions during the dressings.

The prognosis of Bart's syndrome depends on many factors such as the severity and extent of ACC, epidermolysis bullosa subtype, associated abnormalities and the effectiveness of treatment.^3^ This prognosis is generally favorable in high‐income countries.^3^, ^15^ However, in sub‐Saharan Africa, treatment options remain limited and the mortality rate is high. As in our case, among the ACC cases involving the lower limbs described in sub‐Saharan Africa, 4/5 patients (80%) died. Deaths in Africa are linked to complications (anemia^6^), associated congenital abnormalities (possible congenital heart disease^8^), limited financial means, and insufficient technical equipment.

## 4 CONCLUSION

We hereby report the first Cameroonian case of Bart's syndrome associated with disorder of sexual differentiation, which ultimately progressed to death due to severe anemia. Bart's syndrome remains a rare disease, mainly diagnosed clinically. In some cases, it can be associated with birth defects. The prognosis is generally good if the conditions for adequate management are met.

## CONFLICT OF INTEREST

The authors declare no conflict of interest.

## AUTHOR CONTRIBUTIONS

Odette Berline Sigha involved in data collection, writing of the initial manuscript, and revision of the manuscript for final submission. Ritha Mbono Betoko and Mireille Fossi Happi involved in data collection and revision of the article. Grace Anita Nkoro, Benjamin Bertrand Kelbaba, and Charlotte Eposse Ekoube involved in revision of the article. Edgar Mandeng Ma Linwa contributed to English translation. Emmanuel Armand Kouotou involved in supervision and revision of the article.

## CONSENT

Written informed consent was obtained from the patient to publish this report in accordance with the journal's patient consent policy.

## Data Availability

The data that support the findings of this study are available from the corresponding author upon reasonable request.

## References

[1] Muoki A , Gatinu BW , Mutiso VM . Aplasia cutis congenita. A case report and review of literature. East Cent Afr J Surg. 2013;18(2).

[2] Mukhtar‐Yola M , Mshelia L , Mairami AB , et al. Aplasia cutis congenita: a report of two cases from National Hospital Abuja, Nigeria and review of the literature. Pan Afr Med J. 2020;17(36):291. 10.11604/pamj.2020.36.291.24523 PMC7572678 33117485

[3] Alfayez Y , Alsharif S , Santli A . A case of aplasia cutis congenita type VI: Bart syndrome. Case Rep Dermatol. 2017;9(2):112‐118. 29033814 10.1159/000478889 PMC5624250

[4] Friedan U . Aplasia cuties congenital: a clinical review an proposal for classification. J Am Arad Dermarol. 1986;14(4):646‐660. 10.1016/s0190-9622(86)70082-0 3514708

[5] Bart BJ , Gorlin RJ , Anderson VE , et al. Congenital localized absence of skin and associated abnormalities resembling epidermolysis bullosa. A new syndrome. Arch Dermatol. 1966;93:296‐304. 5910871

[6] Ogundipe KO , Kadiri IA , Oluwayemi IO , et al. Aplasia cutis congenita: dilemma of management in a resource‐limited scenario. J Surg. 2020;5:1274.

[7] Ahogo KC , Kouassi KA , Cissé L , et al. Aplasie cutanée congénitale et épidermolyse bulleuse (syndrome de BART): une association inhabituelle sur peau noire. Aplasia Cutis Congenita and Congenital Epidermolysis Bullosa (Bart Syndrome): An Unusual Associattion on Black Skin. Rev Int Sc Méd –RISM. 2017;19(1):78‐80.

[8] Oriji PC , Afolabi SA , Omietimi JE , et al. Aplasia cutis congenita: uncommon finding of two cases occurring in one patient in two successive deliveries. Yen Med J. 2019;1:61‐65.

[9] Mava Y , Yakubu AM . Aplasia cutis congenita in a Nigerian child: a case report. Niger J Paediatr. 2017;44(1):32.

[10] Rajpal A , Mishra R , Hajirnis K , et al. Bart’s syndrome. Indian J Dermatol. 2008;53:88‐90. 19881996 10.4103/0019-5154.41655 PMC2763726

[11] Wingi O , Cappellesso R , Arego R , et al. Failed conservative management of a case of aplasia cutis congenita in a low‐income country. Clin Case Rep. 2016;4(8):756‐758. 27525077 10.1002/ccr3.611 PMC4974421

[12] Witchel SF . Disorders of sex development. Best Pract Res Clin Obstet Gynaecol. 2018;48:90‐102. 29503125 10.1016/j.bpobgyn.2017.11.005 PMC5866176

[13] Hughes IA , Nihoul‐Fékété C , Thomas B , et al. Consequences of the ESPE/LWPES guidelines for diagnosis and treatment of disorders of sex development. Best Pract Res Clin Endocrinol Metab. 2007;21(3):351‐365. 17875484 10.1016/j.beem.2007.06.003

[14] Mazen I , Amin H , Kamel A , et al. Homozygous mutation of the FGFR1 gene associated with congenital heart disease and 46, XY disorder of sex development. Sex Dev. 2016;10:16‐22. 27055092 10.1159/000444948

[15] Kulalı F , Bas AY , Kale Y , et al. Type VI aplasia cutis congenita: Bart's syndrome. Case Rep Dermatol Med. 2015;2015:549825. 26609453 10.1155/2015/549825 PMC4644546

